# Health Care Professionals’ Understandings of Cross-Cultural Interaction in End-of-Life Care: A Focus Group Study

**DOI:** 10.1371/journal.pone.0165452

**Published:** 2016-11-23

**Authors:** Anna Milberg, Sandra Torres, Pernilla Ågård

**Affiliations:** 1 Palliative Education & Research Centre, Linköping University, Norrköping, Sweden; 2 Department of Advanced Home Care and Department of Social and Welfare Studies, Linköping University, Norrköping, Sweden; 3 Department of Social and Welfare Studies, Linköping University, Norrköping, Sweden; 4 Department of Sociology, Uppsala University, Uppsala, Sweden; Universidad de Valladolid, SPAIN

## Abstract

**Objective:**

The academic debate on cross-cultural interaction within the context of end-of-life care takes for granted that this interaction is challenging. However, few empirical studies have actually focused on what health care professionals think about this interaction. This study aimed to explore health care professionals’ understandings of cross-cultural interaction during end-of-life care.

**Methods:**

Sixty end-of-life care professionals were recruited from eleven care units in Sweden to take part in focus group interviews. These interviews were analyzed using qualitative content analysis.

**Results:**

The health care professionals interviewed talked about cross-cultural interaction in end-of-life care as interaction that brings about uncertainty, stress and frustration even though they had limited experience of this type of interaction. The focus group discussions brought attention to four specific challenges that they expected to meet when they care for patients with migrant backgrounds since they took for granted that they would have an ethno-cultural background that is different to their own. These challenges had to do with communication barriers, ‘unusual’ emotional and pain expressions, the expectation that these patients’ families would be ‘different’ and the anticipation that these patients and their families lack knowledge. At the core of the challenges in question is the idea that cross-cultural interaction means meeting “the unknown”. In addition, the end-of-life care professionals interviewed talked about patients whose backgrounds they did not share in homogenizing terms. It is against this backdrop that they worried about their ability to provide end-of-life care that is individualized enough to meet the needs of these patients.

**Conclusions:**

The study suggests that end-of-life care professionals who regard cross-cultural interaction in this manner could face actual challenges when caring for patients whose backgrounds they regard as “the unknown” since they anticipate a variety of challenges and do not seem confident enough that they can provide good quality care when cross-cultural interaction is at stake.

## Introduction

There is evidence of health inequalities and poorer quality of health care and outcomes among people from minority ethno-cultural and linguistic backgrounds [1–3].This is also true in the context of end-of-life care [4–5]. Moreover, literature on what it is like to offer care over cultural, ethnic, linguistic and religious boundaries has shown that interaction between health care professionals and dying patients who do not share a common ethno-cultural background can be challenging [5–16].

As a response to these inequalities and challenges, it is sometimes assumed that the best way to manage cross-cultural interaction is to raise health care professionals’ cultural competence (e.g. [4–5] [11] [17]). However, there is no agreement about what cultural competence is or how it should be taught, practiced or evaluated [16–18]. In addition, theories on culture competent care have been criticized (e.g. [5–6][13] [17] [19–20]) because they tend to stress the importance of patients’ ethno-cultural backgrounds and disregard the relevance of other background factors (such as gender, age and class). With regard to palliative care specifically, critics have suggested that we should be skeptical of the cookbook-like approaches of the various adaption models that culture competent care theories have generated since such guidelines may create a multitude of myths and stereotypical images of patients with minority ethno-cultural backgrounds, ‘‘routinize” interactions between care professionals and patients with such backgrounds and impede holistic care [13] [16] [20].

Thus, even though there have been numerous efforts to find effective ways of addressing the inequalities and challenges mentioned earlier, there is a lack of understanding of the complex processes involved in cross-cultural interaction during end-of-life care. Research has so far tended to focus on the ways in which these interactions are experienced retrospectively as opposed to the understandings that precede them, and only a few studies have gone beyond the care professionals’ experiences in such meetings, e.g. [15]. This means that the challenges associated with cross-cultural interaction are reduced to being about how things ‘are’ (according to an individual’s previous experiences) as opposed to how individuals expect them to be. In other words, the fact that one’s expectations of such interaction can have an impact on how the interaction is experienced is not acknowledged. This is why this study focuses on the ways in which cross-cultural interaction is understood. By ‘understandings’ we mean the ways in which care professionals regard this interaction, irrespective of whether or not they have actually experienced it. Therefore, in this study our aim was to explore health care professionals’ understandings of cross-cultural interaction during end-of-life care.

### Context of the Study

Sweden is a country with an ethno-culturally diverse migrant population which is why cross-cultural interaction within health care settings has been on the agenda of care planners and providers for some time, e.g. [21]. During 2012 (the year when the data was collected for this study), Sweden had 9,555,893 inhabitants of which 15.4% (1,473,256) were foreign-born. Roughly 103,000 persons immigrated to Sweden and 43 887 sought asylum that year [22]. In 2012, more than half of the foreign-born came from a European country, and about 30% arrived from an Asian country. The most common country of birth was Finland (about 11%), and thereafter came Iran and Poland [23]. During 2010–2015, the number of persons seeking asylum more than doubled in Sweden [24]. The ethno-cultural diversity of Sweden’s population combined with the fact that Swedish research on health disparities focusing shows that people with migrant backgrounds in this country have poorer self-reported health than native-born individuals [25] and the fact that research has shown that Swedes tend to regard its migrants as ‘the Other’ [26] means that the provision of care to patients with such backgrounds in this country is on the agenda of the health care sector. The sector worries that it will not be able to meet the ‘special needs’ with which a migrant and ethno-culturally diverse minority population is associated [27] which is why patients with migrant backgrounds are often singled out when cross-cultural interaction in the context of health care provision in this country is being discussed. It is against this backdrop that we deemed the context in question to be an appropriate site to study to explore health care professionals’ understandings of cross-cultural interaction in end-of-life care.

## Methods

### Participants

In the present study, the participating health care units had a variety of different focuses (internal medicine (n = 2), surgery (n = 3), geriatrics (n = 2), specialized palliative home care (n = 2), primary care (n = 2)), but all had to provide end-of-life care as one of their assignments. [Inclusion criteria for participants: health care staff member at a participating unit. Exclusion criteria: No experience regarding care of dying patients.] There was no requirement that the participant had own experience of cross-cultural interaction since the study focuses on care professionals’ understandings of this phenomenon as opposed to their experiences of it. We used a combination of maximum-variation sampling and convenience sampling [28]. Table 1 provides the sample characteristics for this study and gives us a glimpse at what lies behind the understandings that the analysis have unveiled. Worth noting is also (as Table 1 shows) that the informants experience of caring for patients with the backgrounds in question was limited.

**Table 1:** Sample characteristics (n = 60).

Female/ Male (n)	58/ 2
Age in years (median (range)/ not responded (n))	47 (23–64)/ 2
Staff member’s profession: nurse/nursing assistant/ doctor/ occupational therapist (n)	39/ 19/ 1/ 1
Experience of caring for dying patients of ethnic minorities: Extensive experience/ Some/ Non / not responded (n)	4/ 45/ 10/ 1
Work place (n): Specialised palliative home care/ Non-specialised palliative care^a^	16/ 44
Years of experience in the profession (median (range) (n))	16 (0.5–44)
Years of experience in end-of-life care (median (range)/ not responded (n))	17 (1–44)/ 1

^a^internal medicine, surgery, geriatrics or primary care.

### Data collection and analysis

The informants received written information (authored by the researchers, distributed by the head of the unit) and oral information about the study (from the researcher who visited the units at a staff meeting). This information stated clearly that the study focused on cross-cultural interaction and would bring attention to what their thoughts about caring for patients with migrant and ethno-cultural minority backgrounds. Those interested in participating contacted the head of the unit, who then, along with the interviewer (the third author), organized the focus group interviews (cf. [29–30]). Previous to the focus group interviews, the informants were asked by the interviewer to fill out a form with general information about themselves (the information in Table 1 stems from that form) and to give written consent. The interviews were conducted in a secluded room at the units where the professionals worked (or close to them). They were semi-structured, were based on an interview guide [31] and included open-ended questions. The first question we posed had a general focus (i.e. “What associations do you get when you think of caring for dying patients with migrant/ ethno-cultural minority backgrounds?”). The questions that followed were more specific and focused on cross-cultural interaction using a chronological design (e.g. “What associations do you get when you think of the first meeting with the patient?”; “… the further care of the patient?”; “… the care during the very last phase of the patient’s life?” “… when the patient dies?”; “… the care after the patient has died”). A similar design was used when posing questions about interaction with the family and/or relatives of patients with such backgrounds. Since there is no agreed definition of end-of-life care in practice [32], we were inspired by Gysels et al.’s work and used two approaches [32] aiming to arrive at the participants’ understandings of cross-cultural interaction during end-of-life care by both, in relation to time (exemplified by questions relating to care when the patient is dying and cure is no longer possible), and by approaching the scope of end-of-life care in an integrative way through the use of WHO’s definition of palliative care when we asked: “We now would like you to think of the WHO’s definition of palliative care, where symptom relief in a broad sense is included, as well as communication, team work and family and tells us what you think about it [33]”; e.g. “What associations do you get when you think of symptom relief in a broad sense (i.e. physical, psychosocial and spiritual) to dying patients of ethnic minorities?”). Follow-up questions were asked when needed [30]. Dialogical validation of the informants’ statements was performed during the interviews by re-phrasing and checking whether they had correctly understood the questions [34]. The duration of the focus groups interviews ranged 47–89 minutes; median 70 min.

A qualitative latent content analysis was performed on the collected data [35] using the computer software NVivo 10. The analysis was performed according to the following steps: (1) the transcribed focus group interviews were read through to obtain an overall impression (naive reading); (2) The material was re-read carefully to identify text segments that were laden with meaning (meaning units), i.e. text segments where the health care professionals described some aspect of understandings of cross-cultural interaction during end-of-life care; (3) The meaning units were condensed and abstracted to preliminary codes; (4) The codes were then compared and sorted into categories and sub-categories from which a broader theme was developed. A theme is considered to be a thread of an underlying meaning on an interpretative level, that is, the latent content of the meaning units, codes or categories, [35]; (5) The categories, sub-categories and theme were compared to the full text material in order to check that the interpretation was consistent and coherent with the interviews as a whole, and that the meaning had not been changed during the analysis; (6) The categories, sub-categories and theme were compared to prevent overlapping, and content descriptions were developed of the final categories, sub-categories and theme; (7) The findings are exemplified by using quotations.

The development of the first preliminary coding scheme was mainly carried out by the third author. This coding scheme and the analysis performed were examined by the other two authors through peer-debriefing sessions [36], the aim of which was to ensure the trustworthiness of the analysis. Thus, all authors participated in the analysis of the data in order to reduce the risk of investigator bias and to increase reflexivity [28] [35] [37]. The inter-professional team of three researchers consisted of one associate professor who has scientific and clinical experience as a palliative care consultant as well as experience in qualitative methodology (the first author); a professor of sociology whose research focuses on ethno-cultural relations and has vast experience in qualitative methodology (the second author) and a master level student in sociology (who is now working toward a Ph.D. that focuses on the issues at hand) (the third author). The regional board of ethics (”Regionala etikprövningsnämnden i Linköping, Avdelningen för prövning av övrig forskning”) approved the study (Dnr 2011/341-31). The present study is part of a larger project on end-of-life care for patients with migrant background, and a previous publication focused on patient-centredness [38].

### Findings

**Fig 1:**
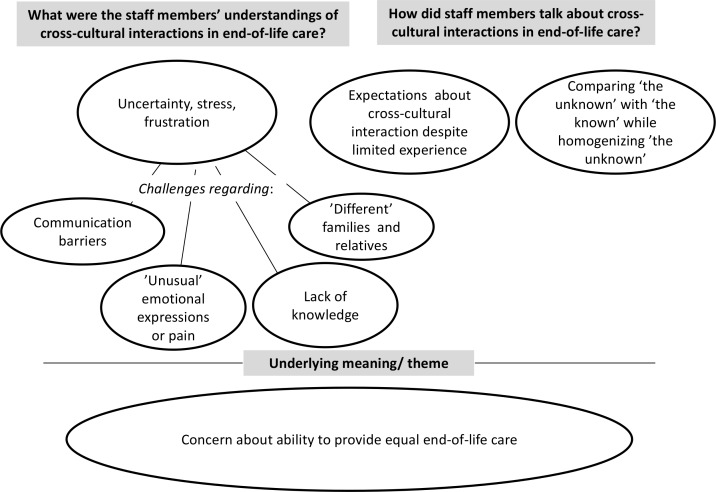
Overview of the findings regarding health care professionals’ understandings of cross-cultural interaction in end-of-life care,

Three categories, four sub-categories and one theme emerged in the analysis of the focus group interviews, see Fig 1.

#### Uncertainty, stress, and frustration

The health care professionals’ understandings conveyed that cross-cultural interactions brought about uncertainty, stress, frustration, ethical dilemmas, and a feeling of not doing a good job. In addition, they had expectations of being questioned and mistrusted in their professional role. The health care professionals interviewed took for granted–just like the academic debate does—that they would face a variety of challenges when involved in cross-cultural interaction within the context of end-of-life care. The following are the challenges that they expected to face when caring for patients with migrant and ethno-cultural minority backgrounds; which are the patients with which they associated cross-cultural interaction.

i) Challenges regarding communication barriers: The health care professionals interviewed associated cross-cultural interaction with communication challenges not only in terms of language barriers (first quotation below), but also in terms of body language and cultural norms regarding appropriate ways to convey different types of emotions (second quotation below).

Well you know it makes things harder if they can’t speak Swedish. Then it’s very difficult… to interpret. And then also… you know getting things across to this severely ill patient who maybe is in the dying phase and well, being able to calm him or her down and talk in the way you usually do [if the patient can speak Swedish], all of those small things that you have inside and say all the time, you know you can’t say them because they won’t understand.

(Focus group interview 9, six participants)

——————

X1 Exactly, the silent language… body language. That we shake hands, say hello, maybe all cultures don’t do that. We touch our patients quite a bit because we often do that in our culture when we want to calm someone down. But that might be completely wrong in another culture, a woman touching a man or a man touching a woman. I think about things like that a lot.

X2 That can make you a little stiff too. You don’t dare get too close because what if I break all those unwritten rules that I don’t know about.

(Focus group interview 8, six participants)

The informants thought that the use of interpreters in end-of-life care was valuable, but mentioned that it was not easy to work with an interpreter since one could not expect them to be present at all interactions that took place with patients. They feared also that using interpreters meant being uncertain as to what was actually communicated to the patient by an interpreter (irrespective of whether they were dealing with an official interpreter or a family member).

Sometimes, family members demanded to act as in that capacity. In these situations, the care professionals worried not only about the quality of what was being interpreted but also about the fact that using family members to communicate sensitive information to the patient about his/her condition could both burden the family member and prevent the patient from being informed about death-related issues.

At the very core of how the challenges associated with communication were talked about was the fact that the care professionals interviewed took for granted that cross-cultural interaction entailed not being able to communicate in the way they were accustomed to. This meant, in turn, that this interaction was expected to be ripped with misunderstandings, uncertainty and frustration. They expected also that inadequate symptom relief could be a given when involved in this type of care interaction.

ii) Challenges regarding ‘unusual’ emotional expressions or pain: The informants thought that patients with migrant and ethno-cultural minority backgrounds have their own ‘unusual’ ways of expressing feelings and it was clear that they regarded the emotional expressions that they associated with the ethnic majority as the ‘norm’. In comparison to them, migrants and ethno-cultural minorities were expected to be louder, show more dramatic ways of expressing pain (and other symptoms), emotions and grief. The quote below illustrates this:

They often show they’re in pain in a different way, so that you think ‘oh boy,’ you compare them with stiff Swedes who are, like, almost dying and only say that it just hurts a little. Compared with someone who’s a bit temperamental, foreign, who really screams when it’s a sore or something that a Swede maybe wouldn’t have mentioned. It’s very hard to interpret so there’s a lot of misunderstanding.

(Focus group interview 11, four participants)

Here we see that when talking about cross-cultural interaction, the health care professionals interviewed described that the family members of ethnic minorities could, for example, faint, scream, break things and threaten staff physically as well as verbally in their frustration and grief. They discussed also the following:

X1 But even if you’re sort of prepared for things to get noisy still sometimes it’s unpleasant.

X2 Yes.

X1 That’s what I think anyway and I stand by it. You know I try to deal with it but well when they’re all running around like chickens with their heads cut off just screaming and yelling and… I mean it’s pretty difficult.

X3 Yes it is.

//…//

X1 They grab the dead body and shake it, you know…

X4 Yeah. It’s quite something; they really act out their feelings.

X? Uh huh.

X3 The enormous sorrow you encounter, it’s so tangible, it really affects you. (Focus group interview 8, six participants)

Cross-cultural interaction was, in other words, discussed as interaction that entails being exposed to expressions of emotions, feelings and pain that can sometimes be so different that they are, in and by themselves, an added, new layer of emotional stress for them. This means that feelings of discomfort, inadequacy, and uncertainty were expected when involved in this type of interaction.

iii) Challenges regarding ‘different’ families and relatives: Patients with migrants and ethno-cultural minority backgrounds were expected to have more family members travelling, sometimes from far away, to visit the ill person in the hospital as well as at home, and were also more likely to have relatives closely involved in the care of the patient. The informants thought that this could be challenging since it meant having more people asking questions, which took up more time.

Compared to what they considered to the norm as far as networks of affiliations are concerned–in this case Swedes—the families of migrant and ethno-culturally minority patients were also expected to have a strong sense of community and to support the patient as well as each other in remarkable ways. This meant that although cross-cultural interaction was assumed to be more time consuming, it could also be less so since highly-involved relatives meant that the staff did not have to professionally intervene as much. However, the informants envisioned that it could be difficult to assess the situation of these patients because it was difficult to get to know them on a one-to-one basis. Moreover, the health care professionals interviewed mentioned that it could be difficult to know with whom in the group of many relatives they were meant to be communicating or who was in need of extra support.

In addition, when the staff talked about this type of challenge, they also mentioned that the interactions with these families tended to be more demanding compared to interactions with average family members of Swedish patients because the staff felt questioned and mistrusted in their professional role. The following quote illustrates this:

X1… there’s this doubt, it’s there I mean, it’s not just their manner but there’s more weight behind it, right. And I think we encounter it now and then actually, more being called into question.

X2 There are often greater demands, and doubt.

X1 Right and not trusting. … a little distrust, are we really doing what we should, have we done everything? Things like that I think.

(Focus group interview 7, four participants).

Here we see that family members of ethnic minorities were expected by the staff to express stronger opinions about what the patient should or should not be informed about in relation to the progressive disease, about the need of an assessment by a doctor (instead of a nurse), and about the gravity of a situation and the need of quick help from health care.

iv) Challenges regarding lack of knowledge: The final type of challenge discussed had to do with these patients’ lack of knowledge, or different points of reference, as far as the end-of-life process and care were concerned. For example, basic knowledge about how palliative care and health care in general was expected to be lacking in these groups (e.g. specific visiting hours, restrictions on bringing food to patients receiving hospital care, the division of tasks between different professions in health care due to the relatively high competence level in nursing staff in Sweden etc.). In addition, cross-cultural interaction was talked about as interaction that entailed caring for patients that lacked a basic understanding of how the body is built and/or works (e.g. basic anatomical knowledge about the main organs, which is needed if one is to understand symptoms and procedures).

X1 They lack knowledge, I mean sometimes their knowledge of the body is very poor //.

X2 ‘Why are you giving the injection here when I have pain there?’ for example. ‘That’s not where it hurts! You have to give me the injection here.’ That’s happened several times. Things like that reduce their trust. ‘She doesn’t know what she’s doing, she’s putting the needle in my arm when my stomach hurts. She doesn’t know anything.’

(Focus group interview 2, six participants)

Challenges associated with lack of knowledge were also about the lack of knowledge that the care professionals themselves felt they had with regard to insight into other cultures and religions (e.g. how to communicate about these issues with ethno-culturally ‘different’ patients and families), and also about ethno-cultural rituals in relation to dying, death and bereavement.

/…/ when it’s things like this that you think are really strange, like tying together the [dead person’s] feet, then you think is this really okay, can we really do this? That’s what I think that I’d think when we’re so unfamiliar, but if you know that, and think yeah I’ve heard about this before, this happened. Well there are, do you see what I mean. That would be easier. (Focus group interview 1, seven participants)

Challenges in relation to lack of knowledge seem to create frustration and uncertainty. In addition, the lack of knowledge they themselves felt they had, was knowledge they felt could easily be conveyed to them and which would make cross-cultural interaction feel less ‘unusual’.

#### Expectations about cross-cultural interaction despite limited experience

As already stated, the study brings attention to understandings of cross-cultural interaction within end-of-life care irrespective of whether or not one has experience of this type of care interaction. Worth noting is, however, that although most of the health care professionals interviewed had limited experience of this type of interaction (as shown in Table 1), they seemed to have very clear ideas about what this would entail and what the challenges they could expect to face would be about. This means that the focus group discussions were carried out as if they knew what this type of care interaction was like and how it would feel. Thus, although, fifty five out of the sixty participants stated that they had some or no experience of cross-cultural interaction, there was little hesitation in their discussions about what this interaction would entail within the context of end-of-life care. Most of the discussions in the focus groups were, in other words, carried out as if experience was a given amongst them, and we noted on numerous occasions that when we actually asked our informants if they had experienced the challenges that they were describing at length, they would say that they had not. Thus, during the focus group discussions it was very common for the informants to describe vividly and in detail their expectations of this type of interaction after having first stated: “I never cared for someone with an immigrant background, but…”(Focus group number 5, seven participants).

#### Comparing ‘the unknown’ with ‘the known’ while homogenizing ‘the unknown’

The analysis showed also that the health care professionals interviewed tended to compare patients with migrants and ethno-cultural minority backgrounds with Swedish patients when describing what they associated with and/or expected from cross-cultural interaction. This means that the focus group discussions were ripened with comparisons between what they regarded as ‘familiar’ and/or ‘the norm’ (i.e. patients with Swedish backgrounds) with what they expected to be ‘unusual’ and/or ‘different’ (i.e. patients with migrant and ethno-cultural minority backgrounds). The first and second quote alluded to in the section on challenges regarding communication barriers and the first quote used in the section on challenges regarding ‘unusual’ emotional expressions or pain are examples of this.

Moreover, when talking about cross-cultural interaction within end-of-life care, the health care professionals interviewed did not bring attention to specific ethno-cultural backgrounds but talked about the patients in question in homogenizing ways. They seemed, in other words, to pay limited attention to specific ethno-cultural backgrounds per se (which would have entailed expressing concern about the heterogeneity of backgrounds encompassed by the ethno-cultural diversity that characterizes this particular context) and talked instead of patients with migrant and ethno-cultural minority backgrounds in homogenizing ways (i.e. they talked, for example, about patients from “another culture” or referred to them as “they” and contrasted them with patients who they considered to be similar to them (i.e. patients from “our culture”; “Swedes” or “us”). The first quote used in the section on challenges regarding ‘unusual’ emotional expressions or pain as well as the first quote used in the section on lack of knowledge are examples of this. It is against this backdrop that we suggest that the understandings of cross-cultural interaction that are upheld by the health care professionals interviewed are informed by a juxtaposition of ‘the known’ to ‘the unknown’ which builds on the homogenization of ‘the unknown’.

#### Concern about ability to provide equal end-of-life care

The health care professionals interviewed agreed that palliative care (which they understood in terms of appropriate symptom management, supporting quality of life for the dying patient, as well as supporting patients’ family members), should be accessible to all irrespective of their ethno-cultural backgrounds. However, they expressed concern about their ability to provide equal end-of-life care to people with migrant and ethno-cultural minority backgrounds because they considered these patients to be ‘different’ and seem to regard similarity of backgrounds as a pre-requisite for the provision of good quality of care. The first and second quotes used in the section on challenges regarding communication, the first quote used in the section on ‘unusual’ emotional expressions or pain, and the first and second quote alluded to in the section on lack of knowledge are all examples of this.

Worth noting is also that their concerns seemed to be based on the fact that they expected to encounter difficulties in managing the symptoms of dying patients with migrant and ethno-cultural minority backgrounds because they expected they would have a hard time understanding these patients’ expressions of pain and/or other symptoms. According to the care professionals interviewed, equal end-of-life care requires that staff and patients share similar expectations of the care to be provided and of the ways of handling the end-of-life process. Thus, the informants took for granted that providing care to patients that come from “another culture” (and who have different expectations of care) would be challenging and seemed to doubt their ability to meet these patients’ needs. It is because of this that they claimed that they should be given knowledge about ethno-cultural minorities and that failure to give them this knowledge could jeopardize their ability to meet the challenges they expected to face.

## Discussion

This article sheds light on health care professionals’ understandings of cross-cultural interaction in the context of end-of-life care in Sweden by bringing attention to the expectations that these professionals have of what the provision of care to patients with migrant and ethno-cultural minority backgrounds could be like. Some of the expected challenges that this study brings attention to (i.e. communication barriers, ‘unusual’ emotional expressions or pain, ‘different’ families and relatives, as well as lack of knowledge), and the consequences of such challenges in terms of staff feeling uncertainty and stress are supported by previous research [5–8] [10] [19]. One of the identified challenges entailed the notion that these patients’ and their families lack knowledge on an array of issues. The patient’s alleged lack of “basic” knowledge in terms of anatomy, physiology, medical treatment, self-care etc. has not been discussed as a challenge in cross-cultural care interaction Irrespective of what lies behind these expectations, it must be noted that previous research has revealed not only that there is a paucity of information in minority languages and formats, but also that there may be a lack of cultural equivalents (or negative connotations of such equivalents) of some of the concepts that health care professionals use during end-of-life care (e.g. ‘‘palliative,” ‘‘hospice,” and ‘‘patient burden”) [5].

Another identified challenge had to do with the expectation that cross-cultural interaction entailed dealing with families that are ‘different’, and the fact that dealing with these families meant feeling questioned and mistrusted in one’s professional role. In this respect it seems important to point out that health care professionals need to be made aware that patients’ mistrust may be caused by past personal experiences of racism or perceived racist attitudes among staff, or may be part of a more general cultural mistrust of health professionals that originates from racial discrimination, and a history of segregated and inferior care of minorities [5] [11] [39](cf. [40]).

A result we also would like to draw attention to is that most of the discussions that took place in the focus groups tended to be carried out as if experiences of cross-cultural interaction were a given amongst the informants, although the majority of them had in fact limited experience of this type of interaction. Thus, limited experience of cross-cultural care interaction did not, in other words, hinder care professionals from having understandings of what this entails; understandings that were loaded with assumptions regarding the challenges they could expect. How such understandings impact on care professionals’ actual provision of end-of-life care to the groups of patients in question has not been studied here, but we would like to suggest that future research should apply such a process perspective. Focusing on groups as opposed to individuals (which would entail exploring how professionals’ individual understandings of cross-cultural interaction develop in staff groups at units providing end-of-life care to these patients) seem also like an angle worth studying.

It is also worth noting that when the informants discussed cross-cultural interaction, they tended to disregard specific ethno-cultural backgrounds. Previous research has indicated that nurses rarely remember patients’ cultural background, and that cultural knowledge is not always recalled in practice [16]. These findings contrast with the great attention that is given to specific ethnic backgrounds within culture-competence models, and also with the “culture knowledge” that our informants wished they had as a way of improving end-of-life care to ethno-cultural minorities. The interviewed health care professionals’ lack of attention to patients’ specific ethno-cultural backgrounds may be interpreted as either a result of not recalling (but in the situation paying attention to the ethno-cultural ‘differentness’ of the patient), or actually disregarding ethno-cultural background per se (and equating ethno-cultural background with ethnic minority background, irrespective of the specific ethno-cultural origin). The latter would suggest a more generalized attention to difference in line with the identified pattern we have named ‘comparing the unknown to the known while homogenizing the unknown’. This too seems like an angle worth exploring in the future in relation to education and research. These results point, in other words, to the need to reflect upon the implications that the vast heterogeneity in individual ethno-cultural backgrounds [6] [19] could have on the provision of end-of-life care. The results suggest also that health care professionals need to nuance their understandings of what their own ethno-cultural background (i.e. “the known”) could mean to cross-cultural interaction and should be encouraged to reflect upon the implications that their expectations could have as far as their reactions to “the unknown” are concerned.

The challenges that the interviewed health care professionals expected seemed to contribute to their anticipation of stress in cross-cultural interaction. They seem also to make them doubtful of their ability to meet the needs of patients with migrant and ethno-cultural minority backgrounds. The care professionals interviewed took for granted that equal provision of end-of-life care and treatment based on individualized needs required that care professionals and patients shared similar expectations of the care to be provided and/or had similar ways of handling the end-of-life process. The notion of equity was formulated in terms of fulfillment of the WHO’s goals of palliative care [33] that were used in the interview guide; goals that the health care professionals interviewed seemed very familiar with. However, these goals as well as several other central concepts within palliative care, e.g. good death, are not global and culturally neutral, but have ethno-cultural underpinnings [14] [19] [32]. Therefore, it seems relevant that education in end-of-life care incorporates discussions about the expectations and challenges with which cross-cultural interaction can be associated.

### Limitations

A few issues ought to be noted in terms of the transferability and limitations of the findings. First of all, it must be pointed out that the data was gathered through focus group interviews as opposed to individual interviews. This could be considered a problem but research on focus groups suggests that collecting data through group discussions is most appropriate when wanting to obtain information about professional practice since such practice is often decided upon through informal talk between peers, and focus groups are particularly fruitful when trying to bring such talk to light [41]. Focus groups are also known to be particularly propitious for interviews that require informants to open up about practices that they are uncertain about and/or issues that are highly debated and are, as such, deemed to be controversial [30]. Although we strived to get a range of the phenomenon that was as broad as theoretically possible (i.e. maximum-variation sampling), there was an element of convenience sampling (i.e. involving the selection of the most accessible subjects) [28]. The fact that most health care professionals interviewed were women should therefore be noted as a limitation even though the gender distribution of our sample reflects the actual situation in many health care units. In addition, we did not collect systematic data of how many of the interviewed health care professionals had ethno-cultural minority backgrounds themselves. We take for granted, however that such backgrounds were rare amongst the interviewees. Thus, although we agree that a purposeful sampling of health professionals from such backgrounds would add new angles of study, this is not something we were able to do in this project due to the demographics of health care professionals in the areas where the data was collected. Finally, during the analysis of the focus group interviews we identified a pattern in the participants’ discussion of cross-cultural interaction where they tended to disregard specific ethno-cultural backgrounds. Since the participants were not asked systematically to reflect upon specific ethnic minority groups, we cannot rule out that the phrasing of the questions in the interview guide (and the terms migrants and ethno-cultural minority) may have contributed to this pattern.

## Conclusions

Important aspects of health care professionals’ understandings of cross-cultural interaction during end-of-life care were identified, e.g. four expected challenges in such interactions. The study suggests that end-of-life care professionals who regard cross-cultural interaction in this manner could face actual challenges when caring for patients whose backgrounds they regard as “the unknown” since they anticipate a variety of challenges and do not seem confident enough that they can provide good quality care when cross-cultural interaction is at stake. These findings have implications for clinical practice, education and suggest that future research should employ a process approach to the study of these types of understandings.
